# Comparative vector competence analysis reveals differential Tembusu virus transmission efficiency between *Aedes albopictus* and *Culex quinquefasciatus*

**DOI:** 10.1186/s13071-025-07141-y

**Published:** 2026-02-26

**Authors:** Xi Zhu, Rongrong Li, Yutian Huang, Ruidong Li, Sihao Peng, Xin An, Yuxin Yang, Yuanyuan Liu, Yiping Wen, Qin Zhao, Shan Zhao, Fei Zhao, Rui Wu, Xiaobo Huang, Qi-gui Yan, Yi-fei Lang, Yiping Wang, Yajie Hu, Yi Zhen, San-jie Cao, Shun Chen, Senyan Du

**Affiliations:** 1 Research Center for Swine Diseases, College of Veterinary Medicine, Sichuan Agricultural University, Chengdu, 611130 Sichuan China; 2 Sichuan Science-Observation Experimental Station for Veterinary Drugs and Veterinary Diagnostic Technology, Ministry of Agriculture, Chengdu, 611130 Sichuan China; 3 Engineering Research Center of Southwest Animal Disease Prevention and Control Technology, Ministry of Education of the People’s Republic of China, Chengdu, 611130 Sichuan China; 4 Key Laboratory of Animal Disease and Human Health of Sichuan Province, Sichuan Agricultural University, Chengdu, 611130 Sichuan China; 5 Institute of Veterinary Medicine and Immunology, Sichuan Agricultural University, Chengdu, 611130 Sichuan China; 6 Sichuan Center for Disease Control and Prevention, Chengdu, 610041 Sichuan China

**Keywords:** Tembusu virus, Vector competence, *Culex quinquefasciatus*, *Aedes albopictus*, Mosquito transmission, Vertical transmission

## Abstract

**Background:**

The Tembusu virus (TMUV) is a mosquito-borne pathogen affecting the birds, causing economic losses in poultry and potential public health risks. Research mainly focuses on virus’s characteristics and its interaction with birds, especially the ducks, while entomological studies monitor mosquito populations for epidemiological insights. TMUV’s transmission dynamics are not fully understood, with varying abilities of mosquito species to transmit the virus. There is limited research on the vector competence and vertical transmission potential of different mosquito species, particularly in China. We compared the vector competence of *Culex quinquefasciatus* and *Aedes albopictus* for TMUV.

**Methods:**

The vector competence of *Culex quinquefasciatus* and *Aedes albopictus* for TMUV was assessed through intrathoracic microinjection and artificial membrane feeding techniques. Mosquitoes were sampled at 1-, 3-, 6-, and 9-days post-inoculation (dpi) following intrathoracic microinjection of TMUV, and at 4-, 8-, and 14-dpi following artificial membrane feeding. Samples of heads, midguts, and salivary glands were collected for subsequent analysis. Furthermore, the potential for vertical transmission of TMUV over two gonotrophic cycles was evaluated using the artificial membrane feeding approach. The distribution of TMUV within various organs and tissues of both mosquito species at 4-, 8-, and 14-dpi was investigated by immunofluorescence assays.

**Results:**

The findings suggest that both *Culex quinquefasciatus* and *Aedes albopictus* are effective carriers of TMUV, with *Aedes albopictus* showing greater vector competence. Vertical transmission of TMUV was observed in both species across two successive oviposition cycles, with *Aedes albopictus* displaying slightly higher efficiency.

**Conclusions:**

This study represents the inaugural comparative assessment of vector competence between Sichuan-native *Culex quinquefasciatus* and *Aedes albopictus* (Guangzhou) for TMUV, thereby addressing critical research gaps and providing novel insights for the development of TMUV biocontrol strategies.

**Graphical abstract:**

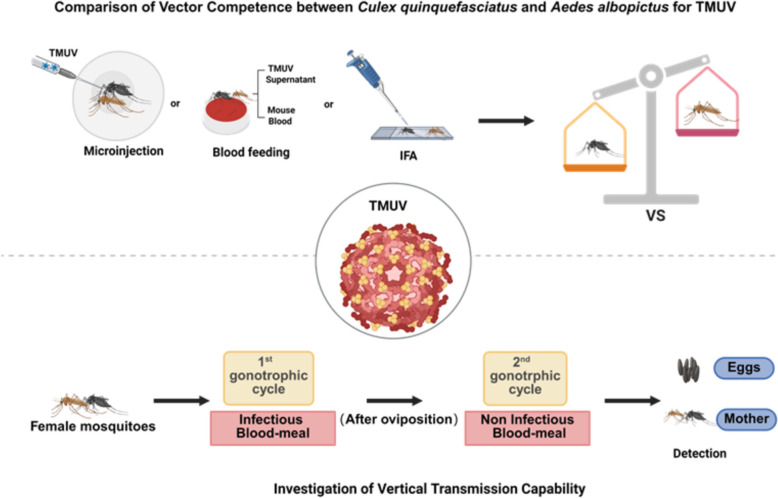

**Supplementary Information:**

The online version contains supplementary material available at 10.1186/s13071-025-07141-y.

## Background

TMUV is a mosquito-borne flavivirus first identified in Malaysia in 1955 and was initially detected in *Culex tritaeniorhynchus* mosquitoes. It was sporadically reported across Southeast Asia during the 1970s [1]. Phylogenetic analyses have revealed three distinct TMUV clusters: Cluster I comprise avian strains from Thailand and Malaysia, Cluster II predominantly contains Chinese avian strains, and Cluster III mainly consists of mosquito-borne strains. Notably, the clustering of mosquito-borne strains with avian strains suggests the potential for a spill-over effect transmission from mosquitoes to birds [2]. Evolutionary analysis revealed that TMUV exhibits a high mutation rate, having expanded rapidly while forming distinct subpopulations and accumulating positively selected sites in the *NS3* and *NS5* genes [3]. TMUV has a broad host range, infecting both mosquitoes and a variety of avian species, including ducks, geese, chickens, sparrows, and pigeons [4–6]. Globally, poultry farming is a vital component of the rural economy, providing both food sources and economic income for local residents particularly in developing countries and impoverished regions [7]. TMUV infection in birds is characterized by sudden anorexia, reduced egg production, and neurological/digestive symptoms [8]. Poultry farms can have infection rates of up to 90%, with 5%–30% mortality among infected chicken flocks [9]. Seroprevalence studies have revealed TMUV-specific IgG or neutralizing antibodies in healthy humans in China and Thailand, although TMUV-positive individuals show no specific symptoms [10, 11]. These findings highlight the significant economic impact of TMUV on poultry farming and its potential as an emerging threat to public health.

In natural ecosystems, the life cycle of most mosquito-borne viruses is a closed loop comprising two stages: vertebrate hosts (such as humans, birds, and pigs) and mosquito vectors [12]. Mosquitoes acquire the virus through hematophagy in infected hosts, after which the virus must overcome several physiological barriers within the mosquito, including the midgut, hemolymph, and salivary gland barriers [13, 14]. Ultimately, the virus reaches the salivary glands, the virus is transmitted to other hosts by subsequent mosquito bites [15]. Mosquito-borne viral disease spread depends mainly on the vector competence of mosquitoes, which varies significantly by mosquito and virus species. For example, *Aedes aegypti* and *Aedes albopictus* serve as the primary vectors for dengue virus (DENV), Zika virus (ZIKV), and chikungunya virus (CHIKV), whereas *Culex tritaeniorhynchus* is the principal vector for Japanese encephalitis virus (JEV) [16, 17]. Despite being a prevalent mosquito species, *Culex quinquefasciatus* has been shown in experimental studies to be an ineffective vector for DENV and ZIKV [18]. Unlike other avian-associated flaviviruses, such as West Nile virus (WNV) and JEV, TMUV relies predominantly on *Culex* mosquitoes as vectors, with *Culex pipiens* and *Culex tritaeniorhynchus* identified as the principal natural vectors for TMUV [19, 20]. The capacity of *Aedes* mosquitoes, specifically *Aedes aegypti* and *Aedes albopictus* (the principal vectors for DENV), to transmit TMUV remains uncertain, although research indicates that *Aedes albopictus* can become infected with TMUV [21].

Mosquito-borne viruses are transmitted primarily through two mechanisms: horizontal transmission, which includes mosquito bite transmission and sexual transmission, and vertical transmission, known as transovarial transmission [22]. The predominant mode of transmission, mosquito-to-animal transmission via bites, is employed by several significant arboviruses, such as JEV, ZIKV, and DENV [23, 24]. Evidence suggests that TMUV also utilizes this mode of transmission. Numerous mosquito-borne flaviviruses are capable of vertical transmission [25]. For example, following the WNV outbreak in California, viral RNA was detected in larvae that hatched from field-collected *Culex* mosquito eggs [26, 27]. ZIKV demonstrates vertical transmission, as laboratory-reared mosquitoes infected through blood meals have been shown to transmit the virus to their offspring, with viral RNA detected in larvae and pupae [28]. Research has shown that TMUV can be transmitted both vertically and sexually in *Culex* mosquitoes [29]. However, research on TMUV transmission mechanisms remains limited, particularly concerning the vector competence of various mosquito species, such as *Aedes albopictus* and *Culex quinquefasciatus*. Furthermore, there is a paucity of information regarding the transmission dynamics, tissue tropism, and molecular mechanisms of TMUV within mosquitoes.

In this study, in vitro membrane feeding and microinjection techniques were used to establish TMUV infection models in *Culex quinquefasciatus* and *Aedes albopictus*. By systematically comparing the vector competence and vertical transmission potential of these two mosquito species, the aim of this research is to assess the vectorial capacity of *Aedes albopictus* for TMUV. Furthermore, the aim of this study was to elucidate the specific differences in TMUV transmission capabilities between *Culex quinquefasciatus* and *Aedes albopictus*, as well as the potential for vertical transmission of TMUV.

## Methods

### Mosquitoes, cells, and virus

*Culex quinquefasciatus* (Sichuan strain) and *Aedes albopictus* (Guangzhou strain) were maintained in mosquito cages at a room temperature of (28 ± 1) °C, a relative humidity of (75 ± 5) %, and under a 10-12 h photoperiod. Adults were given moist raisins or 10% sucrose solution-soaked cotton balls; larvae were fed autoclaved nutrient liver soup (36 g/L liver granules, 24 g/L yeast extract, 36 g/L liver broth, stored at 4 °C). Lab-bred and-acclimated mosquitoes tested negative for TMUV before experiments. BHK-21 and C6/36 cells (from ATCC) were cultured in Gibco’s Dulbecco’s Modified Eagle’s Medium with 10% fetal bovine serum (Excell Bio) and 1% Thermo antibiotic–antimycotic. TMUV (CQW1 strain, KM233707.1) was rescued from infectious clones [30] and propagated in C6/36 cells, with titers assessed via BHK-21 cell plaque assays. Infectious virus experiments were in BSL-2 labs.

### Thoracic microinjection of TMUV in mosquitoes

At 5–7 days post-hatching, female mosquitoes were anesthetized in a cold tray, and the mosquitoes were microinjected with 10 MID_50_/300 nL (5 PFU per mosquito) TMUV for functional studies. The TMUV burden was assessed by RT-qPCR at 1-, 3-, 6-, and 9-dpi. The primers used for gene detection are presented in Table S1.

### Membrane blood feeding

Mouse blood was collected into heparinized tubes and transferred to centrifuge tubes, then centrifuged at 1000 × g for 15 min at 4 °C to separate plasma. The plasma was heat-inactivated at 60 °C for 1 h. The cellular fraction was washed three times with sterile PBS under the same centrifugation conditions, with white blood cells removed each time. The processed plasma and blood cells were mixed and stored at 4 °C. For oral infection, the appropriate amount of TMUV was first mixed with 200 μL of processed mouse blood and serum-free, antibiotic-free DMEM to achieve the desired final viral concentration; the remaining volume was brought up to 800 μL with additional serum-free, antibiotic-free DMEM. The mixture was then placed in a feeder sealed with Parafilm® (Amcor) prior to membrane blood-feeding [31]. Then, 5-day-old female mosquitoes, starved for 24 h, were membrane-fed for 30–40 min. Engorged mosquitoes were transferred to new containers. At 4-, 8-, and 14-dpi, mosquito heads, midguts, and salivary glands were collected for TMUV quantification via RT-qPCR using primers in Table S1.

### Vertical transmission

Experimental mosquitoes were maintained through two gonotrophic cycles per Fig. 5A. In the first cycle, 5-day-old *Culex quinquefasciatus* and *Aedes albopictus* females were fed a 3:1 TMUV-blood mixture via a membrane system. Fully engorged females were individually transferred to separate tubes and maintained at a room temperature of 28 °C and 80% relative humidity for 4 days, after which oviposition was induced by adding water; eggs from each female were collected individually. Post-oviposition females were moved to new containers for the second cycle. After two more days under the same conditions and a 24-h starvation period, females were fed on specific pathogen-free (SPF) BALB/c mice (Vital River) for 30–40 min. Engorged females were isolated and maintained for 4 days. Oviposition was induced again, with eggs and females collected as paired samples. Finally, post-oviposition females were collected into 1.5 mL centrifuge tubes with 300 μL of RNase-free lysis buffer, homogenized, and stored at −80 °C. These samples were later used for TMUV load quantification as described.

### Immunofluorescence analysis

*Culex quinquefasciatus* and *Aedes albopictus* were orally challenged with TMUV at a final blood-meal concentration of 9 × 10^5^ PFU/mL, and samples were collected at 4-, 8-, and 14-dpi. The legs and wings were removed, and the mosquito bodies were fixed overnight at 4 °C with 4% paraformaldehyde. Fixed specimens were submitted to the Baioss Company for paraffin embedding, sectioning (5 μm thickness), immunofluorescence staining, and high-resolution fluorescence scanning. Sections were obtained in the mid-sagittal plane of the mosquitoes. For immunostaining, the sections were incubated with mouse anti-flavivirus envelope (E) protein monoclonal antibody (clone 4G2) (1:200, Genetex) at 4 °C overnight, followed by Alexa Fluor 488-conjugated goat anti-mouse IgG (1:500, Proteintech) for 1 h at RT. Nuclei were stained with DAPI (Solarbio). Fluorescent images were acquired using the Pannoramic SCAN II slide scanner (3D Histech).

### Vector competence indices

Vector competence is assessed via three parameters indicating viral particle progression efficiency in mosquitoes [32]. Infection rate (IR) refers to the proportion of mosquitoes with infected midguts after ingestion of an a virus-containing blood meal, calculated as the number of positive midgut samples divided by the total number of mosquitoes examined, multiplied by 100% [33]. Dissemination rate (DR) reflects the proportion of mosquitoes with midgut infection in which the virus has successfully disseminated into the hemocoel, indicating the efficiency of viral escape from the midgut barrier. It is determined by dividing the number of positive tissue or organ samples (in this study, heads were used) by the number of mosquitoes with infected midguts, multiplied by 100%. A higher DR suggests a weaker midgut barrier. Transmission rate (TR), also referred to as the salivary gland infection rate (SGIR), represents the proportion of mosquitoes with hemocoel infection that have infected salivary glands and are capable of transmission. This metric is calculated as the number of positive salivary gland samples divided by the number of mosquitoes with positive heads, multiplied by 100%. A higher TR indicates a weaker salivary gland barrie [34].

### The establishment of the TMUV-E standard curve

A total of 300 μL of TMUV suspension was used to extract viral RNA following the instructions of the RNA extraction kit (Axygen). Complementary DNA (cDNA) was synthesized using a cDNA reverse transcription kit (Trans Gen). The cDNA was used as a template for PCR to amplify a 404 bp TMUV-E fragment using primers in Table S1. The amplified fragment was recovered via gel extraction kit (Axygen). The extracted TMUV DNA was tenfold serially diluted (10^–1^ to 10^–12^). Real-time PCR was performed on CFX Connect system (Bio-Rad) with SYBR qPCR mix (Trans Gen), using primers presented in Table S1. Each dilution was run in triplicate with blank mosquito samples as negative controls. The mean values of triplicates were analyzed linearly. RT-qPCR templates were aliquoted and stored at −20 °C.

### RNA extraction and reverse transcription qPCR

Total RNA was extracted from mosquito tissues and whole mosquitoes using the Total RNA Extraction Kit (Axygen) following the manufacturer’s instructions. cDNA was synthesized using a cDNA reverse transcription kit (Trans Gen). Quantitative real-time PCR was performed using SYBR qPCR mix (Trans Gen) on a CFX Connect Real-Time PCR Detection System (Bio-Rad). The primers used in this experiment are listed in Table S1, with TMUV-E standards serving as controls.

## Results

### TMUV proliferates more in *Culex quinquefasciatus* than in *Aedes albopictus *via intrathoracic microinjection

**Fig. 1:**
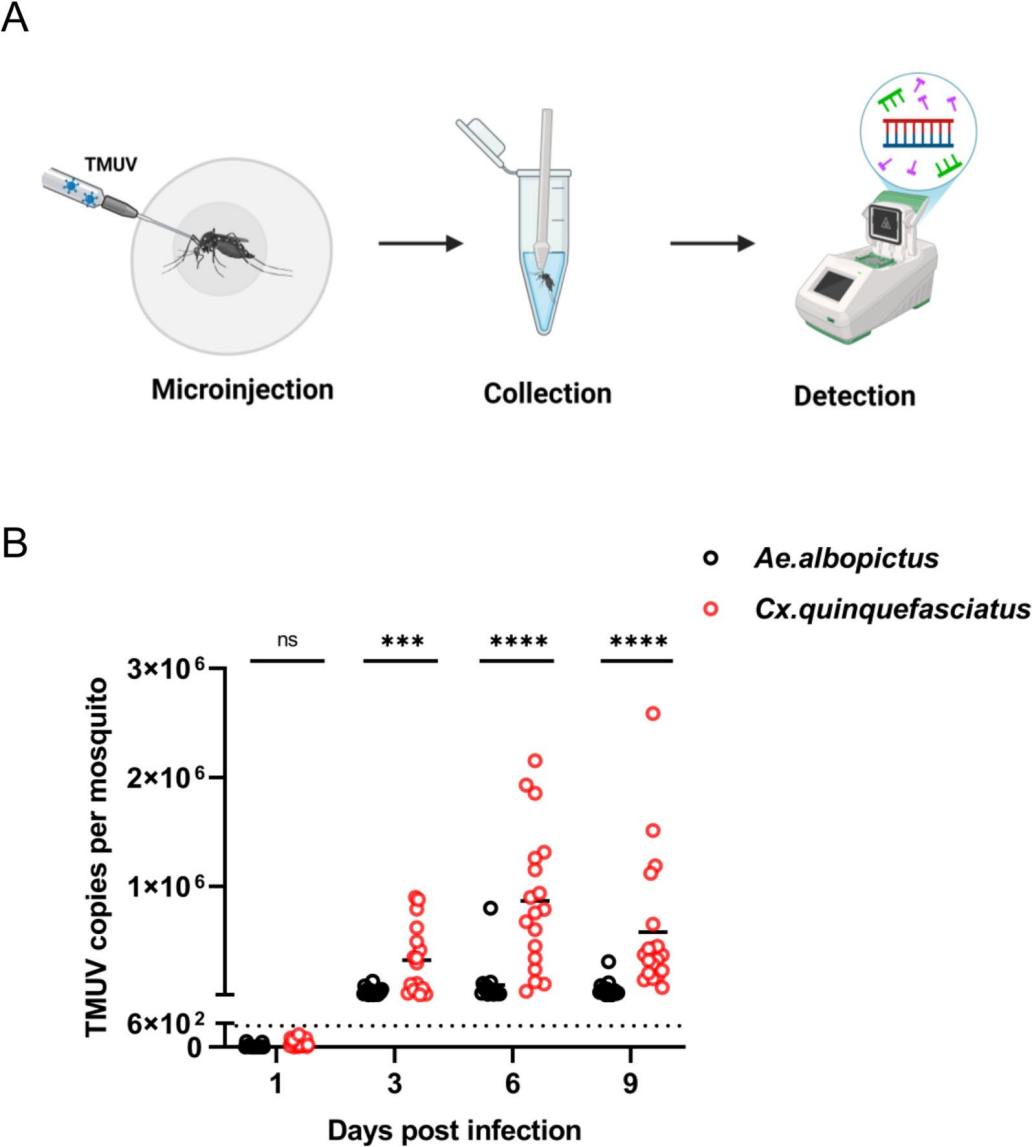
Infection of *Culex quinquefasciatus* and *Aedes albopictus* with TMUV via microinjection. **A** Schematic diagram of the microinjection process. Anesthetized mosquitoes were subjected to microinjection on a workbench. Mosquito samples were homogenized and tested for TMUV, this figure was created with BioRender.com under a paid subscription (Publication License BG28U0SOVQ). **B** Whole-body sampling of mosquitoes at 1-, 3-, 6-, and 9 -dpi was performed, and TMUV levels were detected using RT-qPCR. Each dot represents one mosquito sample, and samples with copy values below 538.6 were considered negative. The horizontal line represents the mean value of the group. Statistical analysis was performed using the nonparametric Mann–Whitney test. n.s., not significant; ***P < 0.001; ****P < 0.0001. The experiments were biologically repeated at least 2 times with similar results, and the data in the figure are from the first experiment

Intrathoracic microinjection reliably induces experimental viral infections in mosquitoes. To compare the viral replication capacities of *Aedes albopictus* and *Culex quinquefasciatus*, we microinjected each mosquito with 5 PFU of TMUV and collected 18 mosquitoes per group at 1-, 3-, 6-, and 9-dpi for analysis; the experiment was performed twice (Fig. 1A).

Figure 1B shows that TMUV was undetectable at 1 dpi but detectable at 3 dpi in both species. Viral replication dynamics differed significantly: in *Aedes albopictus*, the viral load plateaued after 6 dpi, whereas in *Culex quinquefasciatus*, it steadily increased from 3 to 6 dpi and remained significantly greater than that in *Aedes albopictus* at all subsequent time points. These findings indicate that both species are susceptible to TMUV via microinjection, but *Culex quinquefasciatus* has a greater capacity for viral replication.

### *Aedes albopictus* and *Culex quinquefasciatus* are susceptible to TMUV via blood feeding in a dose-dependent manner

**Fig. 2:**
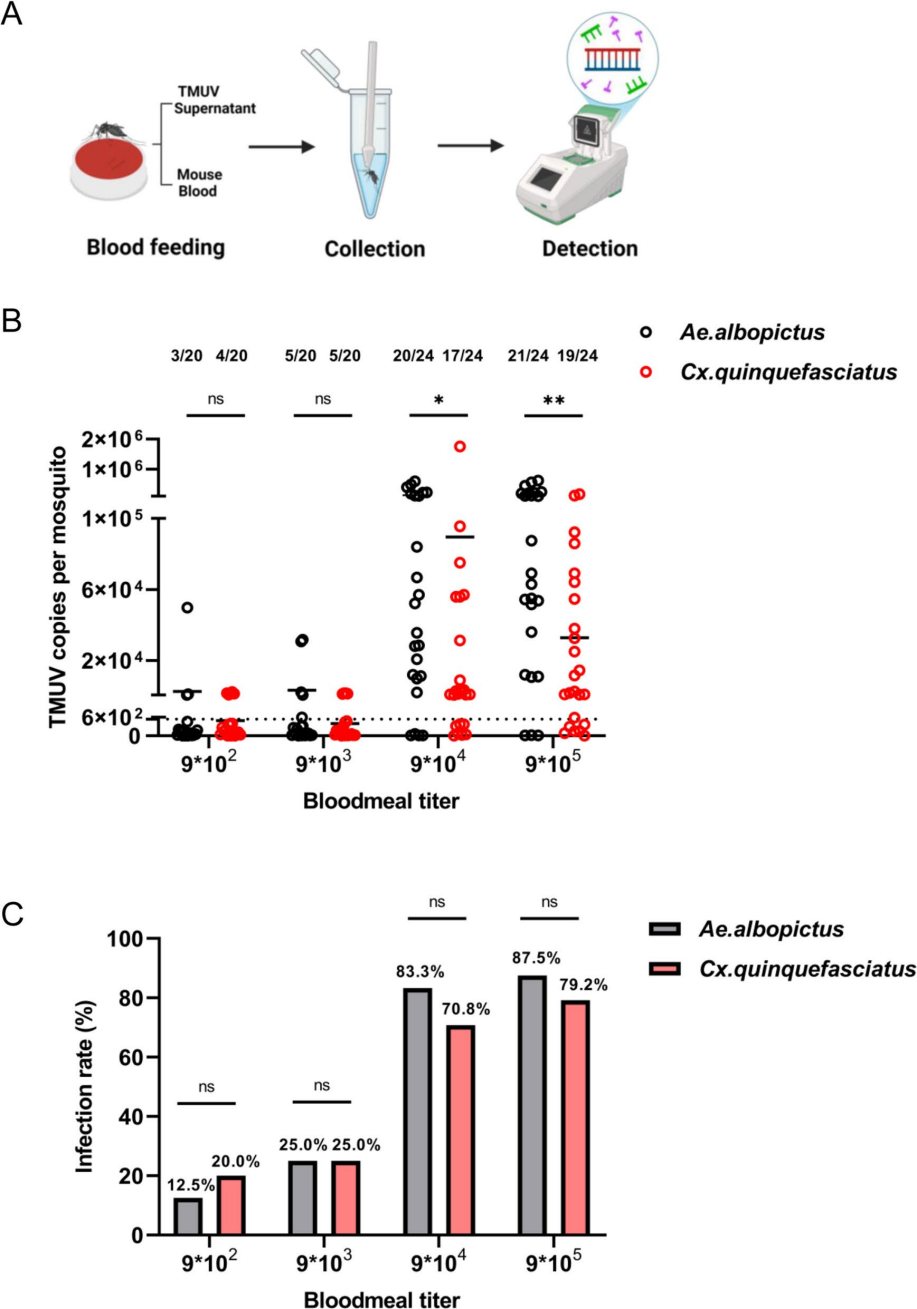
Infection of *Culex quinquefasciatus* and *Aedes albopictus* with TMUV via artificial membrane feeding. **A** Workflow of the artificial membrane feeding method. The feeder was loaded with processed mouse blood and TMUV stock. Mosquito samples were homogenized and tested for TMUV, this figure was created with BioRender.com under a paid subscription (Publication License BG28U0SOVQ). **B** 8 days post-feeding with 9 × 10^2^–9 × 10^5^ PFU/mL TMUV, whole-body mosquito samples were collected, and TMUV loads were detected using RT-qPCR. Each dot represents one mosquito sample, and samples with copy numbers below 538.6 were considered negative. Numbers on the graph indicate positive counts/total samples tested. The horizontal line represents the mean value of the group. Statistical analysis was performed using the nonparametric Mann–Whitney test. n.s., not significant; *P < 0.05; **P < 0.01. **C** Infection rates of TMUV in the two mosquito species under the same conditions. Numbers on each column indicate the TMUV positivity rate. Differences in infectivity ratios were compared using Fisher’s exact test. n.s., not significant. The experiments were biologically replicated at least three times with similar results, and the data in the figure are from the second experiment

Given that viral transmission occurs via blood feeding, we employed an in vitro membrane feeding approach to systematically compare infection outcomes in the two mosquito species. We evaluated the vector competence of *Aedes albopictus* and *Culex quinquefasciatus* for TMUV by administering, through an artificial membrane feeding system, blood meals containing serial tenfold dilutions of the virus at final concentration of 9 × 10^2^, 9 × 10^3^, 9 × 10^4^, and 9 × 10^5^ PFU/mL. Viral loads and body infection percentages were determined at 8 dpi; 20 mosquitoes were sampled per group for the 9 × 10^2^ and 9 × 10^3^ PFU/mL doses, and 24 mosquitoes per group for the 9 × 10^4^ and 9 × 10^5^ PFU/mL doses (Fig. 2A). The results revealed significant differences in viral replication efficiency between the two mosquito species. Quantitative analysis of whole-body viral loads revealed that *Aedes albopictus* exhibited significantly greater TMUV replication than *Culex quinquefasciatus* across all tested viral titers (Fig. 2B). This disparity was consistent irrespective of the initial virus concentration in the blood meal, suggesting a greater susceptibility of *Aedes albopictus* to TMUV infection. Notably, this pattern directly contrasts with our microinjection data shown in Fig. 1B. Both mosquito species presented dose-dependent infection rates, with significantly higher rates observed at elevated viral concentrations (Fig. 2C). For *Culex quinquefasciatus*, infection rates increased from 20% at 9 × 10^2^ PFU/mL to 79.2% at 9 × 10^5^ PFU/mL, whereas *Aedes albopictus* displayed a similar trend, with infection rates ranging from 15.0 to 87.5% (Fig. 2C). Although the viral loads were significantly different, the infection rates of *Aedes albopictus* and *Culex quinquefasciatus* did not show substantial variation in terms of the equivalent virus titers. These findings indicate that both *Aedes albopictus* and *Culex quinquefasciatus* are competent vectors for TMUV, with infection success being positively correlated with the viral titer present in the blood meal.

### *Aedes albopictus* exhibits greater TMUV dissemination and transmission efficiency than *Culex quinquefasciatus* does in laboratory vector competence assays

**Fig. 3:**
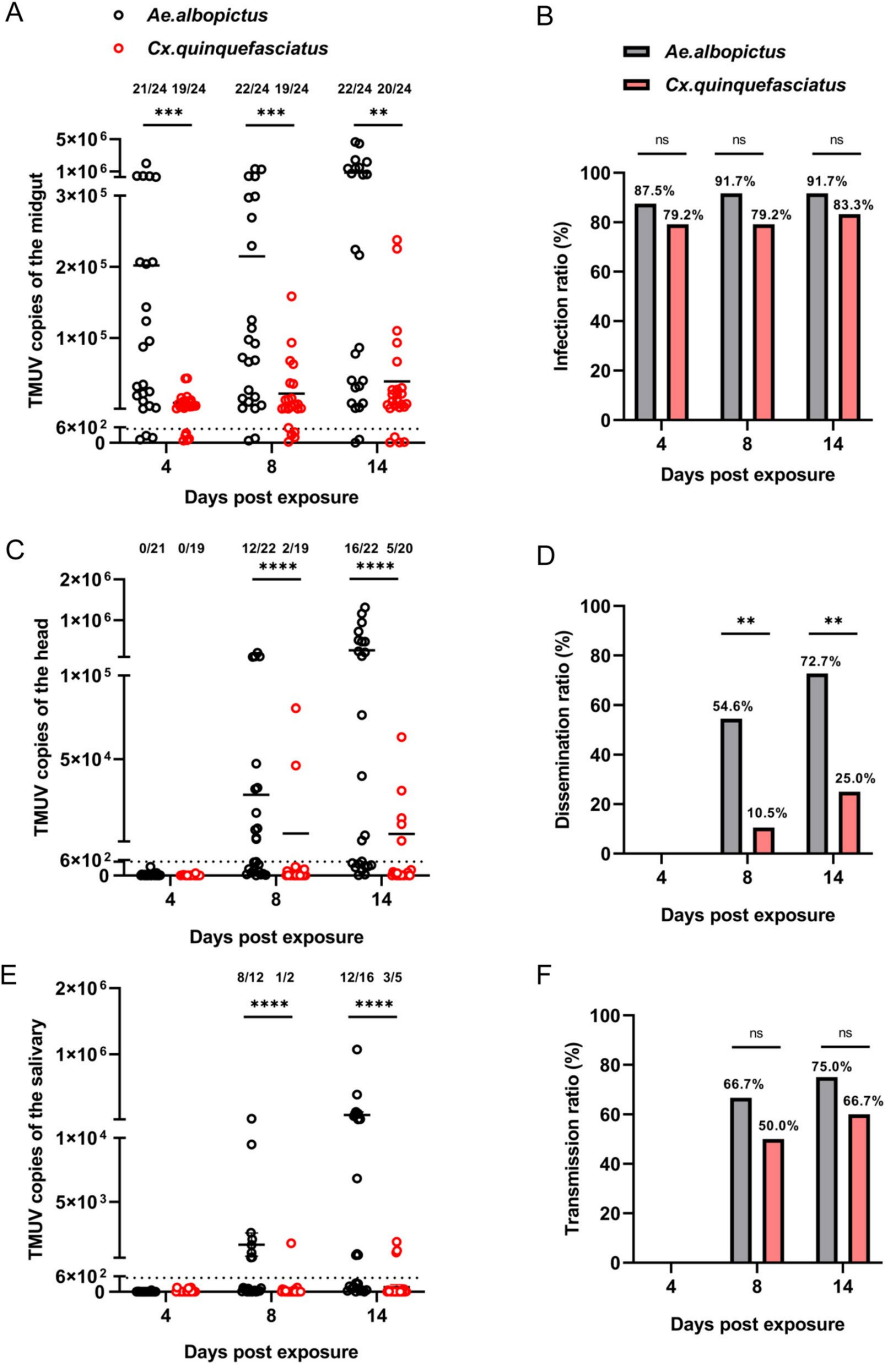
Comparison of the Vector Competence of *Culex quinquefasciatus* and *Aedes albopictus* for TMUV. **A** At 4-, 8-, and 14-dpi with 9 × 10^5^ PFU/mL TMUV, RT-qPCR was used to detect TMUV loads in the midguts of the two mosquito species. Each dot represents one mosquito sample, with copy numbers below 538.6 considered negative. Numbers on the graph indicate positive counts/total samples tested. **B** Midgut infection rates of TMUV in the two mosquito species at 4-, 8-, and 14-dpi with 9 × 10^5^ PFU/mL TMUV. Numbers on each column indicate the TMUV positivity rate. **C** At 4-, 8, and 14-dpi with 9 × 10^5^ PFU/mL TMUV, RT-qPCR was used to detect TMUV loads in the heads of the two mosquito species. Each dot represents one mosquito sample, with copy numbers below 538.6 considered negative. Numbers on the graph indicate positive counts/total samples tested. **D** Dissemination rates of TMUV in the two mosquito species at 4-, 8-, and 14-dpi with 9 × 10^5^ PFU/mL TMUV. Numbers on each column indicate the TMUV positivity rate. **E** At 4-, 8-, and 14-dpi with 9 × 10^5^ PFU/mL TMUV, RT-qPCR was used to detect TMUV loads in the salivary glands of the two mosquito species. Each dot represents one mosquito sample, with copy numbers below 538.6 considered negative. Numbers on the graph indicate positive counts/total samples tested. **F** Transmission rates of TMUV in the two mosquito species at 4-, 8-, and 14-dpi with 9 × 10^5^ PFU/mL TMUV. Numbers on each column indicate the TMUV positivity rate. The horizontal line represents the mean value of the group. The nonparametric Mann–Whitney test was used for statistical analysis of panels A, C, and E. **P < 0.01; ***P < 0.001; ****P < 0.0001. Differences in the infectivity ratio for panels B, D, and F were compared using Fisher’s exact test. n.s., not significant; **P < 0.01. The experiments were biologically replicated at least three times with similar results, and the data in the figure are from the second experiment

To obtain viral dose optimization data, we conducted a systematic comparison of TMUV vector competence between *Aedes albopictus* and *Culex quinquefasciatus* using standardized high-titer infections (9 × 10^5^ PFU/mL) to consistently overcome midgut barriers. This approach enabled us to analyze the dissemination and transmission potential between the two vectors, with 24 mosquitoes sampled per group at each time point. Although both species presented robust midgut infection rates exceeding 79.0% throughout the 14-day observation period, notable differences emerged in their subsequent infection dynamics (Fig. 3A, B). Specifically, *Aedes albopictus* demonstrated significantly greater dissemination efficiency, achieving a 54.6% head infection rate by 8 dpi, which was five times higher than the contemporaneous infection rate observed in *Culex quinquefasciatus* (10.5%). This dissemination advantage became even more pronounced at 14 dpi; 72.7% of the *Aedes albopictus* heads tested positive, whereas only 25.0% of the *Culex quinquefasciatus* heads tested positive (Fig. 3D). Similarly, viral loads in critical tissues consistently demonstrated the superiority of *Aedes albopictus*, with 2.1-fold higher head titers and 3.5-fold greater salivary gland viral loads at peak infection time points (Fig. 3C, E). Notably, the transmission potential quantified by salivary gland infection rates reached 75.0% in *Aedes albopictus*, whereas it reached 60.0% in *Culex quinquefasciatus* by 14 dpi, indicating a distinct advantage in completing the extrinsic incubation cycle (Fig. 3F). Collectively, these findings demonstrate that although both species are capable of acquiring TMUV through blood feeding, *Aedes albopictus* exhibits superior vector competence, which is attributed to its enhanced ability for viral dissemination beyond the midgut and its greater transmission potential.

### TMUV tissue tropism and temporal dissemination

**Fig. 4:**
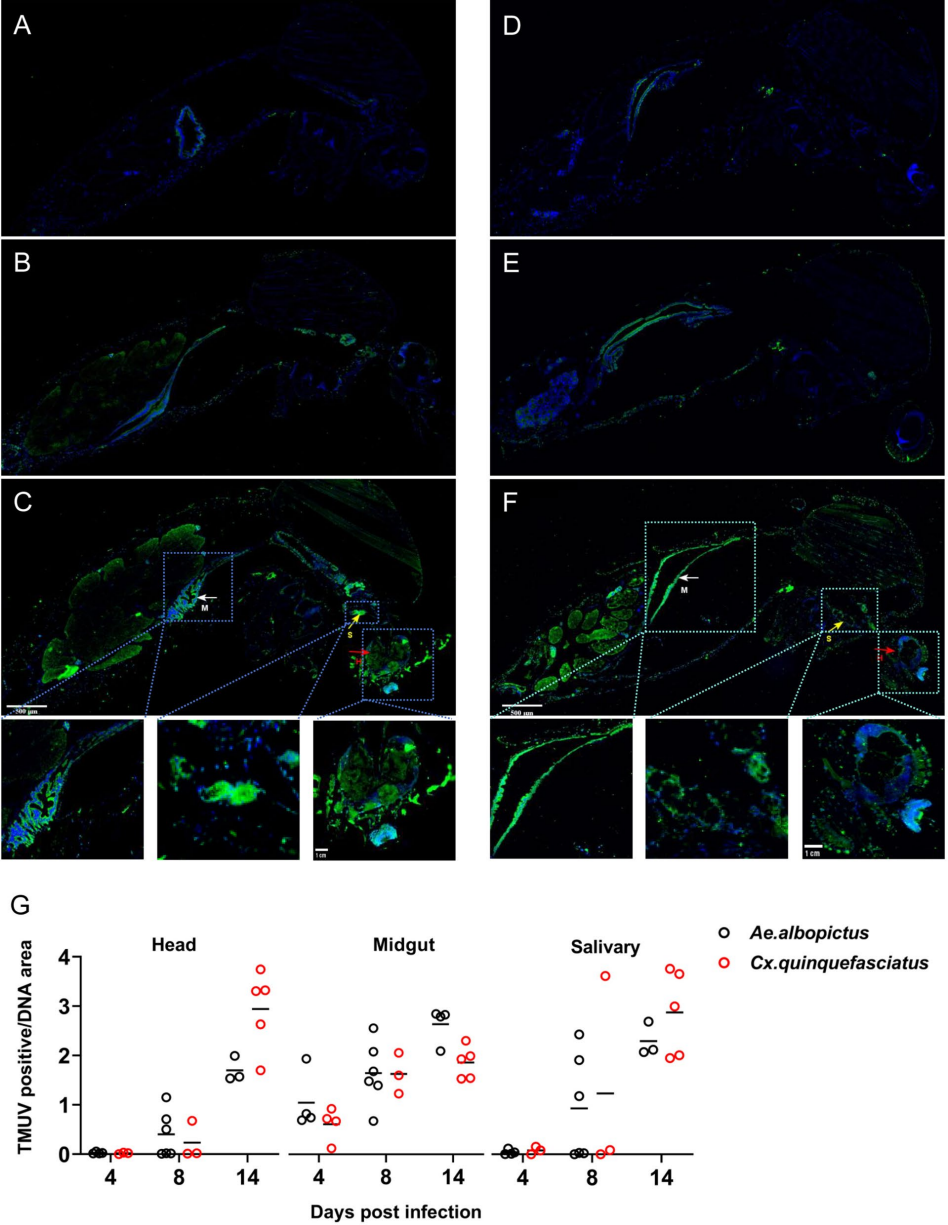
Temporal and spatial distribution of TMUV in *Culex quinquefasciatus* and *Aedes albopictus* mosquitoes detected by immunofluorescence assay (IFA). Mosquitoes were orally infected with TMUV at a final concentration of 9 × 10^5^ PFU/mL via an artificial blood meal. Whole mosquitoes were harvested at 4-, 8-, and 14-dpi and fixed in 4% paraformaldehyde, followed by sectioning and IFA processing. The experiment was independently replicated twice with consistent results; representative images from the second replicate are shown. Uninfected control mosquitoes processed in parallel showed no specific signal. Antibodies used: Flavivirus E protein monoclonal antibody 4G2 (green); nuclei were counterstained with DAPI (blue). **A, B, C** Mid-sagittal sections of TMUV-infected Culex quinquefasciatus at 4, 8, and 14 dpi, respectively. **C**, white arrows indicate the midgut, red arrows the head, and yellow arrows the salivary glands. Insets below (**C**) show magnified views of the midgut, salivary glands, and head at 14 dpi. **D, E, F** Mid-sagittal sections of TMUV-infected Aedes albopictus at 4-, 8-, and 14-dpi, respectively. **F**, white arrows indicate the midgut, red arrows the head, and yellow arrows the salivary glands. Insets below (**F**) show magnified views of the midgut, salivary glands, and head at 14 dpi. **G** Quantification of TMUV-specific fluorescence intensity. The ratio of TMUV E (green) fluorescence area to DAPI (blue) area within specific tissues was calculated using ImageJ. Data points represent individual mosquitoes from midgut, head, and salivary gland tissues across 4-, 8-, and 14-dpi, for both species. Data are pooled from two independent experiments. The X-axis indicates days post-infection, and the Y-axis represents the relative accumulation of TMUV E signal.

To visualize the spatiotemporal distribution of TMUV, we conducted immunofluorescent antibody staining on tissues from *Aedes albopictus* and *Culex quinquefasciatus* over a temporal gradient via a monoclonal antibody specific to the *Flavivirus* E protein. The density of TMUV staining was quantified by measuring the relative stained area within individual organs and tissues by imaging analysis. The dynamics of infection revealed a distinct temporal progression. TMUV was exclusively detected in the midgut of both species at 4 dpi, with localized staining indicating initial viral invasion and replication, Age-matched female mosquitoes that had not received TMUV challenge served as negative controls; the experiment was performed twice (Fig. 4A, D). As the infection progressed, TMUV staining became apparent in tissues or organs adjacent to the midgut beginning at 8 dpi, indicating successful escape from the midgut (Fig. 4B, E). Additionally, it was detected in the heads from 8 dpi. By 14 dpi, TMUV demonstrated extensive tissue tropism, successfully colonizing all major organs of the mosquitoes (Fig. 4C, F). The staining density in the midgut increased significantly, reaching peak levels at 14 dpi. Staining of the salivary glands revealed that a portion of the glands showed infection by 8 dpi. By 14 dpi, viral signals in the salivary glands intensified significantly, indicating active viral replication and accumulation in key transmission-related tissues (Fig. 4G).

### Transovarial transmission dynamics of TMUV in two competent mosquito vectors

**Fig. 5:**
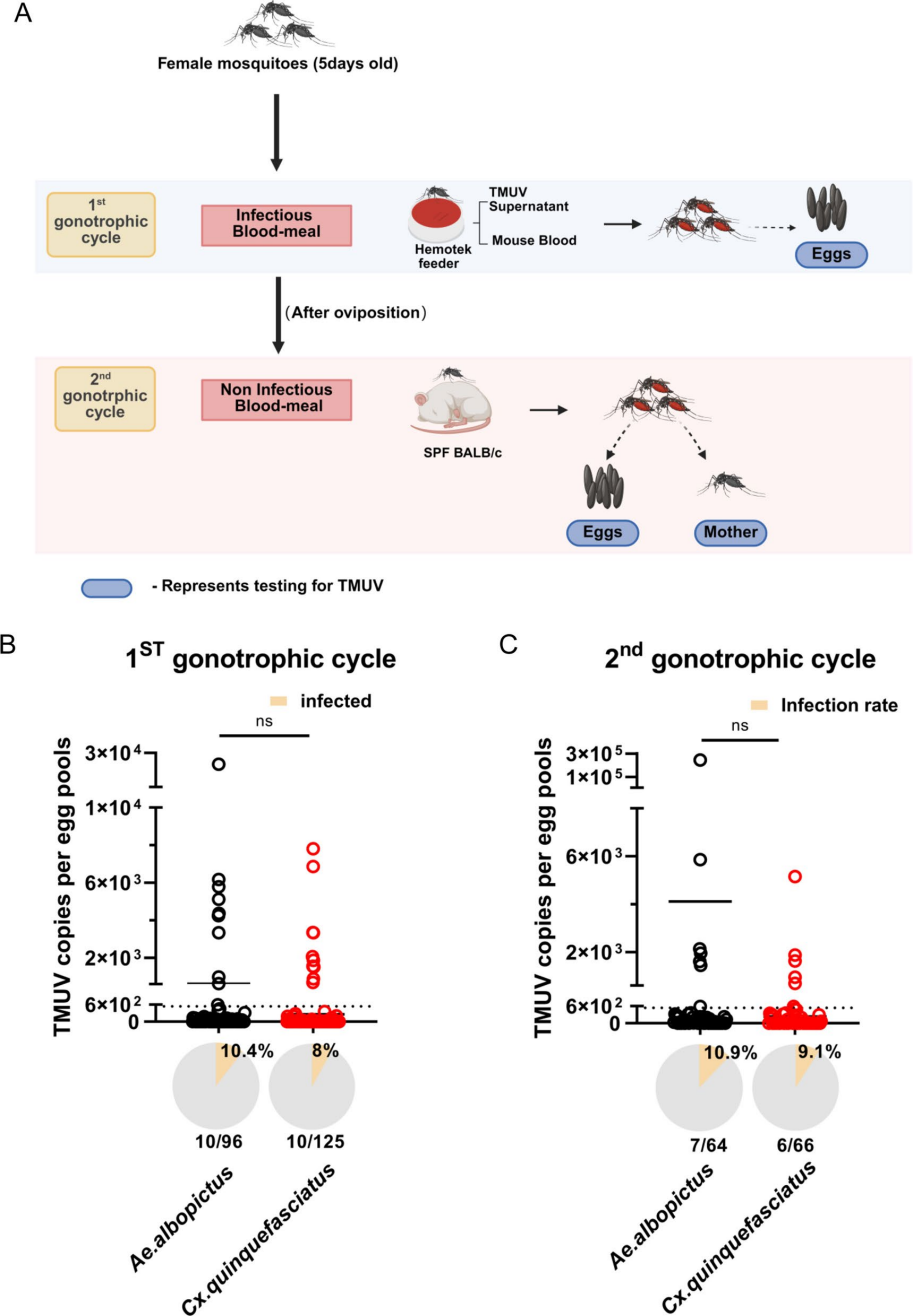
Vertical Transmission of TMUV in *Culex quinquefasciatus* and *Aedes albopictus.* **A** Schematic diagram of the vertical transmission study. Two gonotrophic cycles were conducted. In the first cycle, mosquitoes were fed with infectious TMUV blood, and eggs from this cycle were collected. In the second cycle, female mosquitoes were fed with non-infectious blood, and both eggs and females were collected, This figure was created with BioRender.com under a paid subscription (Publication License XR28U0SDHX). **B** TMUV infection in eggs from the first gonotrophic cycle. Each dot represents one egg sample, with copy numbers below 538.6 considered negative. Numbers on the graph indicate positive counts/total samples tested. Pie charts show infection rates (%), and numbers represent positive eggs/positive female mosquitoes in the first cycle. **C** TMUV infection in eggs from the second gonotrophic cycle. Each dot represents one egg sample, with copy numbers below 538.6 considered negative. Numbers on the graph indicate positive counts/total samples tested. Pie charts show infection rates (%), and numbers represent positive eggs/positive female mosquitoes in the second cycle. The horizontal line represents the mean value of the group. The nonparametric Mann–Whitney test was used for statistical analysis. n.s., not significant. The experiments were biologically repeated at least two times with similar results, and the data in the figure combine the results of the first and second experiments

On the basis of our findings of efficient horizontal transmission, we explored the potential for vertical transmission of TMUV in mosquito vectors, which is critical for viral persistence between epidemics. Using the established infection model (9 × 10^5^ PFU/mL blood meal), we monitored transovarial transmission across two gonotrophic cycles in *Culex quinquefasciatus* and *Aedes albopictus*. The experimental workflow is illustrated in Fig. 5A. Data pooled from two independent trials are as follows. During the first gonotrophic cycle, 160 samples each of eggs and adult females were collected from *Culex quinquefasciatus*, yielding 10 egg-positive pools and 125 virus-positive females; for *Aedes albopictus*, 120 samples each of eggs and females were examined, with 10 egg-positive pools and 96 positive females. In the second cycle, 88 samples each of eggs and females were obtained from *Culex quinquefasciatus*, of which 6 egg pools and 66 females were positive, whereas 75 samples each of eggs and females from *Aedes albopictus* revealed seven positive egg pools and 64 positive females. Through the examination of eggs from *Culex quinquefasciatus* and *Aedes albopictus* over two gonotrophic cycles, it was established that TMUV can be transmitted vertically in both species. Notably, the rates of TMUV positivity in eggs from *Aedes albopictus* consistently exceeded those from *Culex quinquefasciatus* across both cycles. Specifically, 8.0% (10 out of 125 samples) of *Culex quinquefasciatus* eggs were positive during the first gonotrophic cycle, and 9.1% (6 out of 66 samples) were positive during the second cycle. Conversely, the percentage of positive *Aedes albopictus* eggs was 10.4% (10 out of 96 samples) in the first cycle and 10.9% (7 out of 64 samples) in the second cycle (Fig. 5B, C). The marginally higher transmission rates observed in *Aedes albopictus* suggest that this species may play a more significant role in the epidemiology of TMUV.

## Discussion

In 2010, TMUV precipitated a large-scale outbreak among duck populations in southeastern China, resulting in substantial economic losses and posing a potential zoonotic threat [35]. Although commercial inactivated vaccines against TMUV are currently available, the high mutation rate may undermine vaccine efficacy. Research into the vector competence and transmission of mosquito vectors is crucial for preventing viral dissemination and implementing vector-targeted control strategies. TMUV (strain MM_1775) was first isolated from *Culex tritaeniorhynchus* in Malaysia in 1955 [36], and subsequent studies have identified *Culex* mosquitoes as competent vectors for the virus [19]; meanwhile a study in Thailand found that *Aedes aegypti* and *Aedes albopictus* can transmit TMUV at 10^6^ TCID50/mL without impacting mosquito behavior [37]. The present study is the first in which the vector competencies of Chinese *Culex quinquefasciatus* and *Aedes albopictus* are compared. Membrane feeding assays revealed that both species acquire TMUV infection in a dose-dependent manner, with a minimum effective titer of 9 × 10^4^ PFU/mL, which aligns with prior *Culex quinquefasciatus* susceptibility reports [29]. The results also revealed that *Aedes albopictus* has a relatively high salivary gland infection rate. Microinjection experiments indicated that *Culex quinquefasciatus* has higher TMUV loads than does *Aedes albopictus* when bypassing the midgut barrier, suggesting robust systemic viral replication and a possible suppressive effect on the midgut barrier in *Culex* mosquitoes. Notably, *Aedes albopictus* demonstrated greater viral replication than *Culex quinquefasciatus* via blood feeding, as evidenced by their significantly higher viral loads despite similar infection rates. In conclusion, although both *Aedes albopictus* and *Culex quinquefasciatus* serve as competent vectors for TMUV, the dynamics of viral proliferation are significantly influenced by the route of infection. *Aedes albopictus* presented markedly increased viral loads throughout the body following oral infection via blood feeding, indicating effective midgut escape and systemic dissemination. By contrast, *Culex quinquefasciatus* exhibited greater robust TMUV replication than did *Aedes albopictus*. Given that innate immune function of the mosquito gut is relatively conserved across mosquito species [38], the marked difference in TMUV replication load in their midguts leads us to propose that variations in midgut microbiota may be responsible. Recent studies have demonstrated that sphingolipids produced by *Enterobacter hormaechei B17* within the gut microbiota of *Aedes* mosquitoes inhibit ZIKV transmission by preventing viral membrane fusion [39]; the *Rosenbergiella YN46*, isolated from *Aedes albopictus*, suppresses DENV and ZIKV infection by secreting a glucose dehydrogenase (RyGDH) that acidifies the midgut, thereby inducing irreversible conformational changes in the viral envelope [38]. By contrast, a Pseudomonas *sp*. strain in *Aedes albopictus* secretes lipases LipA and LipB, which inhibit viral membrane fusion with host cells by hydrolyzing glycerophospholipids on the midgut epithelial, thereby reducing both infection and transmission [40]. Investigating whether variations in the gut microbiota modulate TMUV transmission and how interspecies differences in the gut microbiota influence vector competence between *Culex quinquefasciatus* and *Aedes albopictus* constitute promising areas of research. Future experiments are planned to treat both mosquito species with antibiotics to deplete their gut microbiota. Upon subsequent blood feeding infection with TMUV, midgut viral loads will be compared with determine the role of microbiota in this process.

Our investigation into the role of *Aedes albopictus* and *Culex quinquefasciatus* as vectors for TMUV demonstrated that both species are capable of effectively transmitting the virus, with *Aedes albopictus* exhibiting notably greater transmission efficiency. Immunofluorescence analysis revealed TMUV accumulation in the midgut at 4 dpi, followed by viral dissemination to adjacent tissues by 8 dpi and systemic spread, including to the salivary glands, by 14 dpi. These findings further substantiate their status as vectors. Vertical transmission, a crucial ecological mechanism for arbovirus persistence in nature, has been previously documented in *Culex quinquefasciatus* [29]. In our study, this understanding is extended by demonstrating TMUV vertical transmission in both *Culex quinquefasciatus* and *Aedes albopictus* across two successive gonotrophic cycles, with *Aedes albopictus* exhibiting higher egg viral positivity rates. The demonstration of TMUV vertical transmission across two gonotrophic cycles in both *Culex quinquefasciatus* and *Aedes albopictus* underscores its potential as a persistent mechanism for viral survival through mosquito generations. The higher viral positivity in the eggs of *Aedes albopictus* suggests an enhanced competency for this transmission route compared with other species. We posit that this enhanced vertical transmission efficiency in *Aedes albopictus* may be linked to a higher viral tropism for its ovarian tissues or a more effective evasion of the transovarial immune response compared with *Culex quinquefasciatus*. These findings underscore the critical importance of mosquito control, particularly targeting *Aedes albopictus*, in TMUV prevention. The TMUV-*Culex*/*Aedes* infection models we developed offer valuable insights for future control strategies. Community-based mosquito egg monitoring could reduce TMUV control costs and benefit infectious disease management in low-income regions. However, the findings of our study are constrained by the use of an artificial membrane feeding apparatus, which may not fully replicate natural conditions. This design excluded examination of viral acquisition from actual duck hosts, including potential species-specific differences and the influence of dynamic viremia profiles in ducks. Further research is needed to evaluate if the vector competence observed here translates to scenarios where viruses are acquired from duck hosts.

In this study, while primarily focused on *Culex quinquefasciatus* and *Aedes albopictus*, emphasizes the necessity of investigating the vector competence of TMUV in additional species, such as *Aedes aegypti*. Furthermore, given that our experiments employed duck-derived viral strains, it is imperative for future research to examine variations in mosquito transmission capacity on the basis of host origin. The findings derived from laboratory settings require validation through field studies conducted under natural conditions. TMUV transmission cycles are complex, making mosquito control a key preventative measure. The data revealed TMUV dynamics in *Culex quinquefasciatus* and *Aedes albopictus*, indicating that *Aedes albopictus* might have increased vector competence for TMUV. These insights may aid in TMUV prevention and highlight the importance of targeted mosquito surveillance and species-specific interventions. In future studies, these observations should be validated in field populations, and the ways that environmental factors impact transmission efficiency should be assessed.

## Conclusions

This comparative study of *Culex quinquefasciatus* and *Aedes albopictus* vector competence for Tembusu virus (TMUV) suggests that *Aedes albopictus* appears to exhibit enhanced transmission efficiency relative to *Culex quinquefasciatus*, while both mosquito species show potential for vertical transmission across gonotrophic cycles. These observations point to a possibly important role of *Aedes albopictus* in TMUV natural circulation, offering insights for developing targeted control approaches. The infection models and vertical transmission data further indicate potential value in larval surveillance and species-specific interventions for managing TMUV transmission. Additional investigation under field conditions and expanded multi-species research would help refine our understanding of TMUV transmission ecology.

## Supplementary Information

Additional file2.

## Data Availability

All the data generated in this study are included in this published article and its supplementary information files.

## Abbreviations

TMUV: Tembusu virus
DPI: Days post-inoculation
DENV: Dengue virus
ZIKV: Zika virus
CHIKV: Chikungunya virus
JEV: Japanese encephalitis virus
WNV: West Nile virus
SPF: Specific pathogen-free
IR: Infection rate
DR: Dissemination rate
TR: Transmission rate
cDNA: Complementary DNA
