# Supplementary material for: Comparative vector competence analysis reveals differential Tembusu virus transmission efficiency between Aedes albopictus and Culex quinquefasciatus
Source: Parasit Vectors. 2026 Feb 26;19:155. doi: 10.1186/s13071-025-07141-y (PMC13077937; doi:10.1186/s13071-025-07141-y)
Supplement: Supplementary file 1 — Additional file2. [file 13071_2025_7141_MOESM1_ESM.docx]

**Supporting information**

**S1 Table** **Primers used for qPCR and genes cloning.**

S1Table

| Primer | Sequence （5'→3'） | bp |
| --- | --- | --- |
| TMUV-E-F | AATGGCTGTGGCTTGTTTGG | 404bp |
| TMUV-E-R | GGGCGTTATCACGAATCTA |  |
| TMUV-PCR-F | TGAACATGGAGGCTACGGAATT | 207bp |
| TMUV-PCR-R | CATCTCAGCAGTGTAGACGGGAC |  |
